# Genetic, Morphological, and Biochemical Diversity of Argan Tree (*Argania spinosa* L.) (Sapotaceae) in Tunisia

**DOI:** 10.3390/plants8090319

**Published:** 2019-09-01

**Authors:** Marwa Louati, Cuneyt Ucarli, Burcu Arikan, Baraket Ghada, Amel Salhi Hannachi, Neslihan Turgut-Kara

**Affiliations:** 1 Université de Tunis El Manar, Laboratoire de Génétique Moléculaire, Immunologie et Biotechnologie, Faculté des Sciences de Tunis, Tunis 2092, Tunisia; 2 Faculty of Science, Department of Molecular Biology and Genetics, Istanbul University, 34134 Istanbul, Turkey

**Keywords:** *Argania spinosa*, Tunisia, genetic diversity, morphological variability, HRM, fatty acid analysis, EPA, DHA, ISSR

## Abstract

Argan trees are normally endemic to Morocco and Algeria, but hundreds of argan trees exist in Tunisia, some introduced from Morocco and some from unknown origins. The aim of the present study was to evaluate the genetic, morphological, and biochemical diversity of the argan trees in Tunisia. In this study, we used morphometric data collected from vegetative tissue, as well as pomological characteristics related to fruits, stones, and kernels. Genetic variation in 60 trees of Tunisian *Argania spinosa* L. was estimated using inter-simple sequence repeats (ISSRs). Mutation screening and genotyping by high-resolution melting (HRM) was performed to detect delta-6-desaturase (*D6D*) variants in the tested individuals, and finally fatty acid analysis of argan leaves with gas chromatography (GC) was performed. The plant materials used in this study originated from four different sites in Tunisia. Analysis of morphological characteristics showed large variability both within and between the studied collections. The analysis of ISSR polymorphisms gave information about the diversity within and between populations. HRM analysis showed that all 60 argan individuals were grouped into 10 different categories. The results of the gas chromatography analysis showed that the presence of omega-3 fatty acids EPA and DHA was noticeable in some argan leaves.

## 1. Introduction

The argan tree is a thorny evergreen tree and one of the most remarkable species of North Africa, both in part to its botanical and bio-ecological interest as well as its social and medical value. These properties have led, in 1925, to the enactment of a special legislation for this species, which in 1998 was declared a Biosphere Reserve Species by the UNESCO and the Biosphere Program. Argan trees are a multi-purpose tree (oil-agriculture-forestry-pastoral), and oil extracted from argan seeds is rich in unsaturated fatty acids [1]. For this reason, argan is popular for its cosmetic, pharmaceutical, and nutritional uses, and its extraction process provides daily jobs and income for rural population. Additionally, argan trees protect the soil from erosion and help fight desertification, and its woody tissue is hard enough to use for construction and heating purposes. Hundreds of argan trees exist in Tunisia; including those with Moroccan origin as well as others with unknown origin, despite the rarity of the species outside of Morocco. The presence of argan trees in Tunisia is encouraging and even prophetic of long-term economic development. Our research will form part of a program for the characterization, conservation, and enhancement of local forest and fruit resources in Tunisia.

*Argania spinosa* is a diploid species (2n = 24) [2] belonging to the Sapotaceae family. It is both monoecious and allogamous [3]. Argan trees can be found in different cities in Tunisia [Sousse, Sfax, Tunis, Kairaouen, Nabeul (Korbous)], and each site contains hundreds of individuals. Tunisian argans are unique compared to those found in Morocco, and morphological and biochemical characterizations of its ecotypes must be carried out in order to elucidate the level of genetic diversity and population genetic structure for further management and development of conservation strategies [4,5]. The genetic diversity of argan trees in Morocco is known to be high [6,7]. This study will determine whether the level of genetic diversity is the same or lower in different countries. In Morocco, the argan tree aids in the preservation of its local environment, and maintains a socio-economical equilibrium at argan sites. Argan’s vast genetic diversity has allowed it to develop an exceptional resilience to climatic changes, particularly in dry locations [8]. In contrast, many species with low genetic diversity are unable to survive in a changing environment [9]. This study uses multiple strategies to determine the degree of argan’s diversity based on its morphological, biochemical, and molecular characteristics.

*A. spinosa* contains oleic acid (OA, C18:1ω3) and alpha-linolenic acid (ALA, C18:3ω3) which have high nutritional and industrial value due to their considerable presence in many essential fatty acids. Delta-6 desaturase is an enzyme that respectively converts linoleic acid (LA, C18:2ω6) and alpha-linolenic acid (ALA, C18:3ω3) to gamma-linolenic acid (GLA, C18:3ω6) and stearidonic acid (SDA, C18:4ω3) [10]. In general terms, the fatty acid content of organisms varies both between and within species. It is believed that this inter-species variation is a direct result of nutrient availability, level of development, weather conditions, and soil characteristics. In addition, minor genetic variations and meta-dependent parameters [trans-methylation and gas chromatography (GC) conditions of fatty acids] can affect fatty acid profiling [11]. The American Heart Foundation emphasized the importance of regulating dietary fats, highlighting in particular the need for monounsaturated and polyunsaturated fatty acids [12]. Polyunsaturated fatty acids (PUFAs) are not synthesized in the human body, and can only be obtained through the consumption of plants and some marine animals [13].

To quantify fatty acid contents within *A. spinosa* leaf samples were collected from 60 trees and analyzed by GC MS. These results were discussed comparatively with those obtained from ISSR and HRM to assess the industrial usage and taxonomic classification of this species.

The genetic diversity of argan trees in Tunisia has not been investigated so far. In fact, not many studies have looked at the genetic variability of *A. spinosa* in general. The earliest research into the genetics of this species looked at isozyme markers isolated from chloroplast DNA using Polymerase Chain Reaction- Restriction Fragment Length Polymorphism (PCR-RFLP) [6,7]. Later, a study was published that used Random Amplified Polymorphic DNA (RAPD) [14,15], and Single Sequence Repeats (SSR) [15] as well as Inter-Simple Sequence Repeats ISSR Polymorphism [16,17,18] and Amplified Fragment Length Polymorphism (AFLP) [19], and more recently Inter Retrotransposon Amplified Polymorphism (IRAP) markers [20]. On the aforementioned techniques, ISSR presents the most consistent method of gene mapping, and yields the greatest number of polymorphic fragments per primer. Essentially, this method allows for the identification of a high number of loci while using only a small number of primers [21]. ISSR markers have been proven successful for assessing the genetic diversity of many species, including *Argania spinosa* [16,17,18], barbary fig (*Opuntia ficus indica*) [22], *Quercus infectoria* [23], *Cassia fistula* L [24], *Nothapodytes nimmoniana* [25], and *Myrciaria tenella* [26].

The purpose of the present study was to evaluate the genetic, morphological, and biochemical variability of natural populations of *A. spinosa* within Tunisia, in order to develop a preservation management program for this species.

## 2. Results

### 2.1. Morphological Analysis of Trees and Fruits

**Figure 1:**
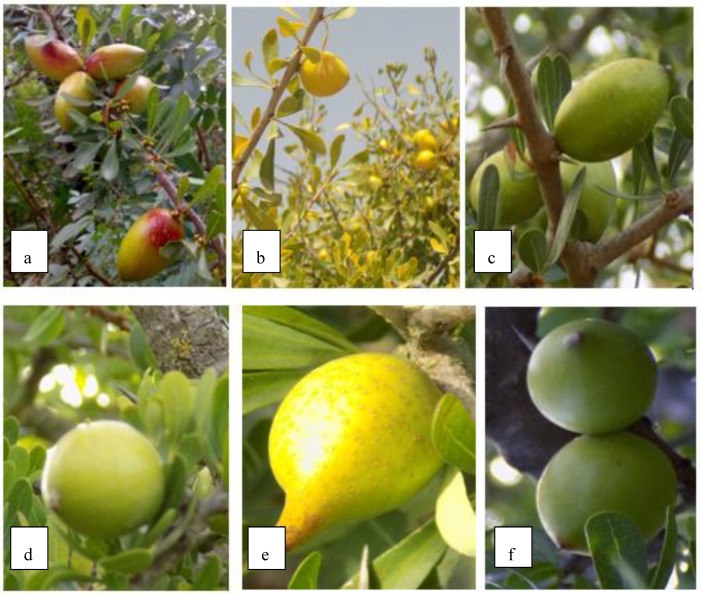
Variability in the shape, color of leaves and fruits (**a**); green and red argan fruits with a fusiform form and dark green leaves, (**b**); yellow argan fruits with an intermediate form and clear green leaves, (**c**); green fruits with an oval form with medium green leaves, (**d**); green fruit with a round form, (**e**); green fruit with an apiculated form, (**f**): green fruits with an intermediate form.

The principal aim of the morphological analysis was to determine the morphological variability of argan between and within the four sites. In this study, a morphological analysis of both vegetative and reproductive tissues collected from 60 individuals across the different sites was conducted (Table 1). Observation of trees from the four sites revealed a high level of morphological variability. Four crown shapes were observed. The frequency of flowers, flower buds, fruits, spines, ramification of plant limbs, as well as colors and fruit maturity were different from one tree to another (Figure 1).

Leaves were observed to be three shades of green, as well as fruits, which came in three colors (green, yellow, and red) in the same period. In addition, five forms of fruit shapes including oval, apiculate, fusiform, round, and an intermediate form were observed (Figure 1).

An analysis of variance (ANOVA) of quantitative measurements collected from fruits and leaves allows for the determination of which variables best discriminate the groups studied. The ANOVA performed on the 22 morphological parameters made it possible to determine the variables that discriminates best individuals from the four sites studied. Many characters present significant differences between the different sites, (*P* < 0.001) indicating high morphological variability between individuals involved in this analysis. The morphological parameters displaying the most variability are primarily those related to fruits and seeds. Some parameters related to kernels and leaves are characterized by a value of F higher than variables that have marked values of *p* > 0.05 and therefore have non-significant differences such as (Lg F, LgG, DG, TG, Nb A, PdA, Lfu, SF) (Table 1).

### 2.2. Principal Component Analysis (PCA)

**Figure 2:**
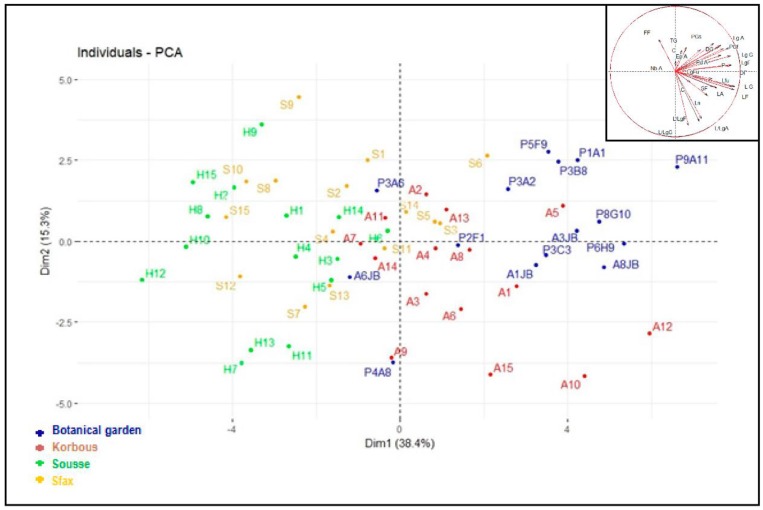
Principal component analysis of different individuals from the four provenances and the circle of correlations of selected quantitative and qualitative variables.

Principal Component Analysis (PCA) was used to demonstrate the variability in a large number of samples with multiple variables [27]. In order to study the morphological diversity between individual argan trees, the averages were taken from various quantitative parameters measured, and used in a principal component analysis. The first component alone absorbed 38.4% of total inertia. It was found that findings positively correlated with each other, such as fruit length, width, and diameter, as well as seed length and width of fresh and dry seed. The second axis absorbs 15.3% of the total variability. It is negatively correlated with fruit size ratio (L/Lg F), seed size ratio (L/Lg G), and kernel size ratio (L/Lg A), and positively correlated with fruit and seed weight. The dispersion of the individuals in the plan defined by the first two components, which absorbed 53.7% of the global inertia (Figure 2), revealed a strong heterogeneity between individuals. Figure 2 shows a clear grouping of argan trees from the two sites; Botanical garden and Korbous, while the other two sites overlapped. Indeed, most individuals inhabiting the Botanical garden and Korbous sites are grouped on the positive side of the axis 1 of this plan. All the individuals from Sousse and most individuals from Sfax were clustered on the negative side of axis 1.

Concerning axis 2, there is an overlap between individuals from all sites (Figure 2). Individuals located on the positive side of axis 1 are characterized by heavier and longer fruits, seeds, and kernels, if compared with individuals positioned on the negative side of the axis. Individuals located on the positive side of axis 2 are characterized by having larger sized (length/width ratios) fruits, seeds and kernels than those positioned on the negative side (Figure 2). A detachment of the individuals P3A6, A6JB and P4A8 was noticed compared to other individuals of the same site on the negative side of axis 1. These individuals are characterized by having the lowest fruit weights (average = 4.6, 4.08, and 4.8 g), seed (2.4, 2.7, and 2.13 g) and kernel (0.3 g) weight in the same site, in addition to having smaller fruits and seeds, which explains its detachment. Another detachment was observed for the individualsA11, A7, and A14 positioned on the negative side of the two axes. These individuals were also characterized by a lower fruit and seed weight, length, and width. A detachment of individuals S14, S6, S5, and S3 was noted compared to argan trees from the same site on the negative side of axis 1. These individuals were characterized by higher fruit, seed, and kernel weights than individuals from Sfax and Sousse (Figure 2). Fruits and seeds from Sfax and Sousse were considered smaller than those from other sites. In conclusion, we found that the projection of the argan trees studied in the plan defined by the first two axes were mainly carried out according to the weight, length, and width of fruits and seeds. Additional parameters tested only contributed slightly to the dispersion of individuals inhabiting the four sites.

### 2.3. Cluster Analysis—CA

**Figure 3:**
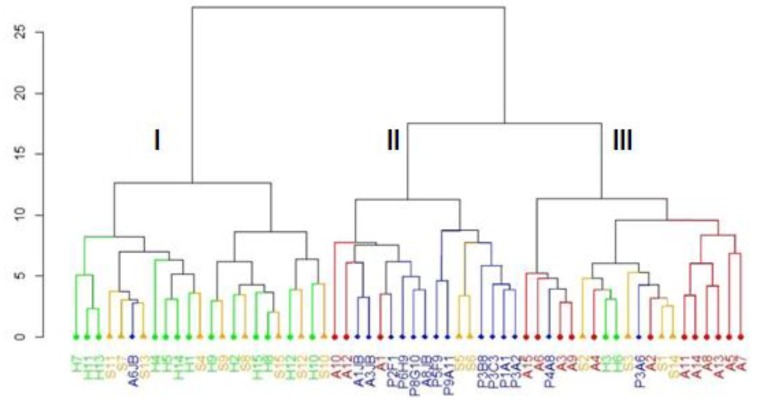
Dendrogram constructed by Euclidean distance using Ward’s method, to study the relationships among the 60 argan trees based on quantitative traits.

The Ward dendrogram was established on the basis of Gower distances. This analysis was based mainly on the quantitative characters since the qualitative characters did not contribute affectively in the PCA. The dendrogram (Figure 3) shows the presence of three major groups (I, II, and III). Group I consists mainly of individuals belonging to the sites Sfax and Korbous, as well as a single individual from the Botanical Gardens (P6JB) which is one of the individual detached in PCA. Group II mainly encapsulates argan trees from the Botanical Gardens, but also contains three individuals from Korbous and two individuals from Sfax. These trees were characterized by having larger and heavier fruits and seeds. The third group is composed mainly of individuals from Korbou, but also contains two individuals from the Botanical garden which are detached in the PCA, as well as two individuals from Sousse and three individuals from Sfax (Figure 3). The results obtained by the analysis of this dendrogram confirm the results obtained by the PCA.

### 2.4. Optimization of the ISSR-PCR Reaction System

In order to optimize the PCR reaction, differentin each primer we played on two variables which are the concentration of magnesium concentrations and the annealing temperatures were tried for each primers. Ten primer pairs were used to produce clear and reproducible fragments. The concentration of magnesium and the annealing temperatures used in this study varied depending on which primers were used (Table 2). Each primer gave a different number of bands that differs from one individual to another. With the number of loci from all individuals we formed a dataset. The 10 primers produced in total 116 bands, in which we obtained 104 polymorphic bands. The percentage of polymorphic loci is 89.66% (Table 3). The size of amplified loci varied from 200 to 2500 bp. Primers ISSR 3/8 and ISSR 807 both showed a lower percentage of polymorphism compared to the other primers (75%) with PIC (Polymorphism Information Content) values respectively 47.6% and 54.6%, while primer ISSR 4/8 showed the highest polymorphism (100%) with a PIC value equal to 66.4% (Table 2).

### 2.5. Genetic Diversity

Among the four argan provenances, the genetic diversity showed that the number of polymorphic loci extend from 90 to 94, and the percentages of polymorphism loci (PPL) range from 77.59% to 81.03%. The observed numbers of alleles (na) were between 1.81 ± 0.39 to 1.77 ± 0.41. The effective numbers of alleles (ne) ranged from 1.41 ± 0.35 to 1.46 ± 0.35 (Table 3).

The range of Nei’s genetic diversity (h) was between 0.25 ± 0.18 and 0.27 ± 0.18. Shannon’s information indexes (I) ranged between 0.38 ± 0.25 and 0.41 ± 0.25. Among the four populations studied, “A” showed the highest genetic variability (H = 0.27; I = 0.41; PPL = 80.17%), while “H” showed the lowest variability (H = 0.25; I = 0.38; PPB = 77.59%) (Table 4).

### 2.6. Genetic Differentiation and Gene Flow

Using Analysis of Molecular Variance (AMOVA), highly significant (*P* < 0.001) genetic differentiation among populations were discovered. Results in Table 5 show that there is 18% genetic differentiation among the provenances, and 82% within the provenances. The Gst coefficient of genetic differentiation was 0.1707. The level of gene flow (Nm) was 2.43, which shows a limited rate of gene exchange among provenances.

### 2.7. Clustering and Genetic Relationships among Argan Provenances

**Figure 4:**
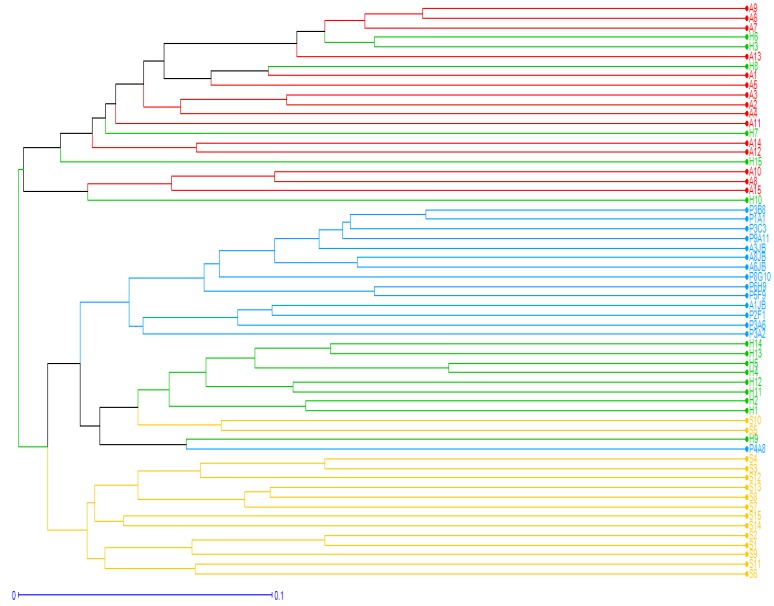
Dendrogram generated by UPGMA clustering analysis using the genetic distance matrix data with 10 ISSR markers, showing the relationship among the four populations of *A. spinosa*. Constructed with Darwin software (boostrap 1000).

**Figure 5:**
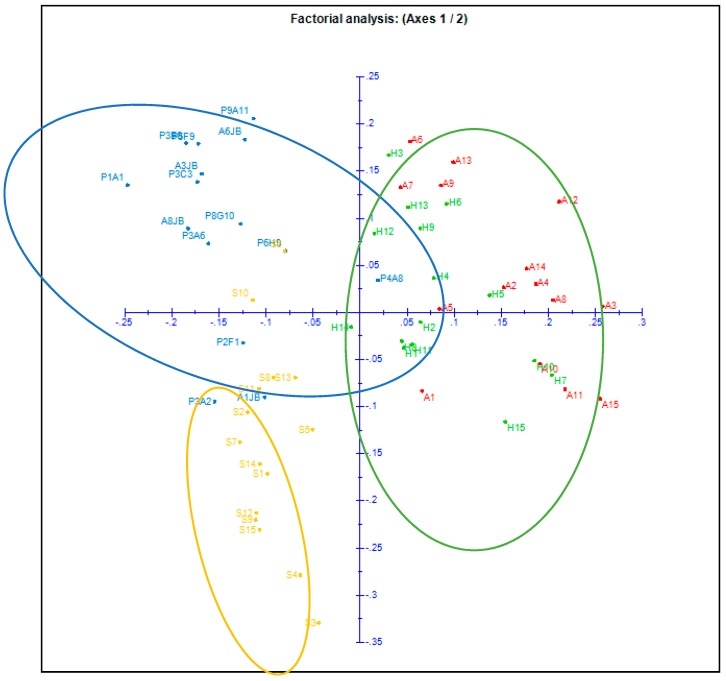
Factorial analysis of different individuals from the four provenances obtained with Darwin software.

The results obtained from Nei’s genetic distances (Table 4) showed that the differentiation among the four provenances ranged from 0.115 between Korbous (A) and Sousse (H), to 0.272 between Botanical garden (JB) and Korbous (A). These findings were confirmed with the unweighted Pair-Group Method with Arithmetic mean (UPGMA) dendrogram which showed that the four provenances formed three major distinct groups of clusters, the first one contained a mixture of individuals from both Korbous (A) and Sousse (H), the second group subdivided into two clear subgroups one containing individuals from the botanical garden (JB), the other containing individuals from Sousse (H) plus one individual from (JB) and two from Sfax (S), while the third group was composed entirely with individuals belonging to Sfax (S) (Figure 4 and Figure 5).

Korbous (A) and Souss (H) were the closest populations and formed a single clustered group, while the majority of individuals from Sfax (S), the rest of individuals from Sousse (H) and the botanical garden (JB) formed single groups except for a few exceptions (S10, S5, P4A8). The relationship among the 60 individuals studied was also demonstrated using Factorial analysis (Figure 5).

The results obtained from these analyses show that individuals were clearly divided into three distinct groups. Using a UPGMA tree (Figure 4 and Figure 5), the first group consists mostly with individuals primarily from (JB) and (H). The second group contains all individuals from (S), while the third contains a mixture of individuals from both sites (A) and (H). Aside from a small detachment of some individuals from (S), and a detachment of one individual, “P4A8” from (JB).

**Figure 6:**
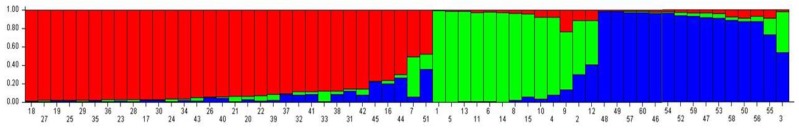
The genetic relationships among the 60 argan tree among the four provenances estimated using the STRUCTURE program based on ISSR data (red: A and H; green: JB; blue: S).

The program STRUCTURE was used to investigate population structure. With the aim of assigning individuals to populations, STRUCTURE was able to confirm the results obtained by using the UPGMA tree and factorial analysis. This program uses a Bayesian algorithm to assess similarities among individuals studied within a known number of genetic groups (or genetic populations, K). Analyzing the results obtained by STRUCTURE using the program Structure Harvester is a fast way to assess and visualize genetic similarities across multiple populations, allowing for the visualization of the number of genetic groups best fitted to the resulting data set. When K = 3, an analysis of assignment probabilities for the 60 individuals studied reveals an increase in cluster resolution. The genotypes were thus grouped into three distinct clusters, the first group (red) contains trees from A and H, the second group (green) contains trees from JB, and the third group (blue) contains individuals from S (Figure 6). S and the JB provenances were observed to be genetically distinct from the two other provenances, A and H which together generated a single cluster (Figure 6.

The assignment of individuals to genetic clusters at K = 3, with the y-axis representing genetic distance and the x-axis the accession and subgroup indicated by color, and with each color representing a single gene pool.

### 2.8. High Resolution Melting (HRM) Analysis

High resolution melting analysis (HRM) is a fast, simple, sensitive, and cheap method for genotyping and mutation screening in variants. The normalized and temperature-shifted difference plots were obtained by HRM assays using the primer pair for the 130-bp region of the Delta-6 desaturase (*D6D*) gene in 60 argan samples. The samples from 60 argan individuals were collected from the four sites studied; Korbus (A), Sousse (H), Sfax (S), and Botanical Garden (JB). HRM classified 60 argan individuals into 10 different groups carrying sequence variances for the *D6D* gene (Table 6). HRM analysis showed that there is a high variability among argan individuals, regardless of proximity to one another.

### 2.9. Fatty Acid Analysis

**Figure 7:**
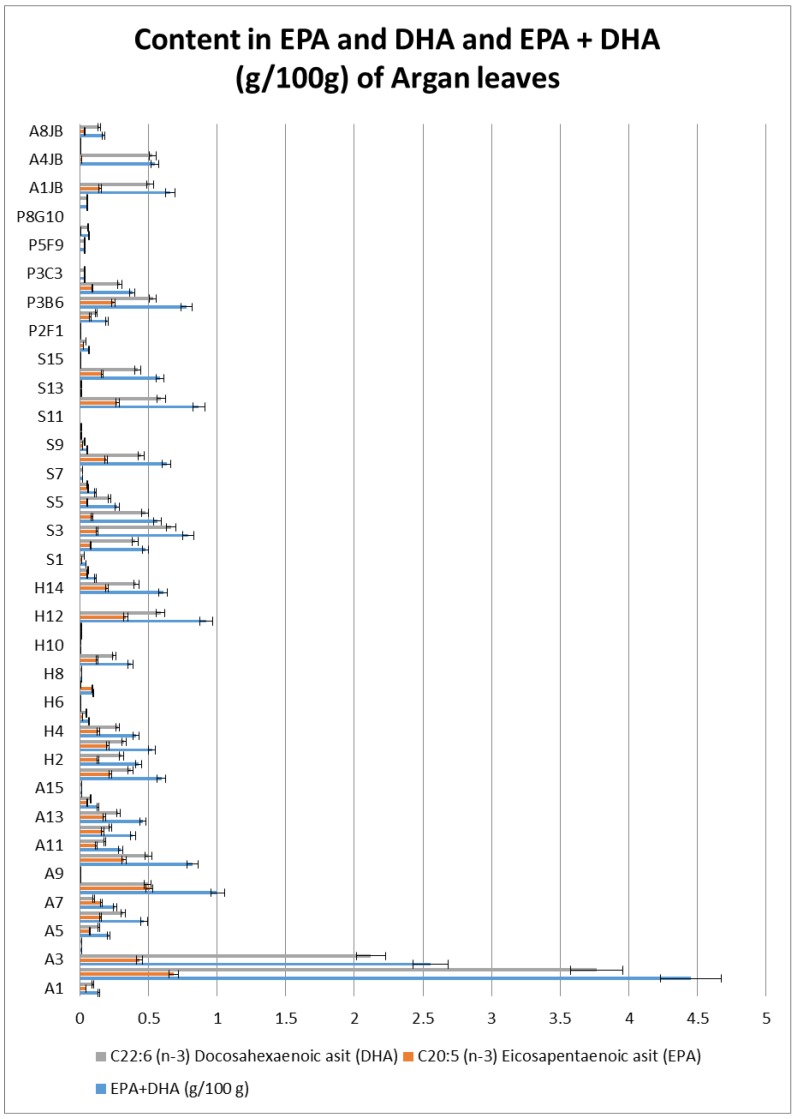
Content of eicosapentaenoic acid (EPA) and docosahexaenoic acid (DHA) and EPA + DHA (g/100 g) in argan leaves.

Fatty acid analysis showed high quantities of eicosapentaenoic acid (EPA) and docosahexaenoic acid (DHA). These are omega-3 fatty acids, which are normally found in cold water fish. EPA and DHA are highly unsaturated fats because they contain six and five double bonds on their long structural chains. The quantities of EPA and DHA vary from one tree to another, some trees are characterized by having high quantities of EPA and DHA while others contain only trace amounts. Individuals containing the highest quantity of EPA and DHA are those inhabiting Korbous; A2 (2.903 g/100 g), A3 (1.631 g/100 g), A8 (1.002), A10 (0.823), while individuals with the second highest quantity of EPA and DHA are found in Sfax S3 (0.788 g/100 g), S4 (0.563 g/100 g), S8 (0.632), S12 (0.866 g/100 g), S14 (0.585 g/100 g) (Figure 7). Figure 7 also shows that the content of DHA in the studied samples is generally higher than the content of EPA.

## 3. Discussion

### 3.1. Morphological Diversity

Argan trees show high levels of morphological diversity both within and between different regions within Tunisia. The variability among trees, such as the morphology of both vegetative and reproductive tissue, is visible to the naked eye. This variability was further highlighted using statistical analysis.

The results obtained from ANOVA show discriminating parameters allowing for the subdivision of argan trees into three groups. Principal component analysis (PCA) results show existence of morphological heterogeneity at both intra- and inter-site levels, taking into account quantitative and qualitative variables. These analyses prove that argan trees display considerable morphological variability. Finally, dendrogram classifying argan trees from the four populations studied indicates the presence of substantial morphological variability at the intra- and inter-site level. This variability may be a result of their geographical origin, as well as the detachment of some of these individuals from their original site. We can conclude that argan trees originating from the four sites studied demonstrate a considerable level of morphological variability at the intra- and inter-site level, which could be a result of the geographical origin of the trees themselves. These observations prove contrary to those obtained by Zahidi [30], whose results indicate great morphological diversity between individuals, independent of their geographical origin. The same results were obtained by Bani-Aameur [31] who carried out a morphological study on 30 argan individuals located in three different sites, based on observations of 11 morphological characteristics. Their results showed there was considerable morphological variability within populations, but there was no considerable diversity. We could explain these results by suggesting a high degree of plasticity within this species.

### 3.2. Genetic Diversity

The degree of genetic diversity between and within populations is the result of a combination of factors such as gene flow, the selection effect, inbreeding, genetic drift, and mutation [32]. When it comes to endangered or at-risk species such as the argan tree, knowing the degree of molecular diversity as well as the genetic structure of this species is an important tool in its conservation [4,5]. Recently, molecular methods have made it easier to analyze the genetic variation of different species, and many studies looking at the genetic diversity of the argan tree have used agromorphological correspondence all observing large phenotypic variability within populations [30,31,33]. In the present study, morphological characterization was performed on argan trees, and showed both inter- and intra- population diversity. However, morphological characteristics are not always enough to conclude the existence of genetic variability. DNA markers have proven to be a precise and reliable method for further analysis of this variability.

In order to adapt to and survive environmental changes, the genetic diversity of a species is vital; the higher the genetic diversity of a species, the higher its chance for survival in a changing climate. ISSRs are efficacious markers used to evaluate polymorphisms as well as the variability of many forest plant species including *Argania spinosa* [16,17,18,34,35,36,37,38,39].

This is the first report of genetic diversity of argan trees in Tunisia. According to the results obtained from this study, argan trees from the provenance Korbus (A), located in the North not far away from the sea, showed the highest genetic variability (H = 0.27; ne = 1.46; PPL = 80.17%), while the provenance Sousse (H) showed the lowest variability (H = 0.25; ne = 1.41; PPB = 77.59%) (Table 3 and Table 4). However, this location is dryer and characterized by a different soil composition. If all individuals were considered to have inhabited the same location, an even higher genetic diversity is noted (ne = 1.53, h = 0.32, PPL = 89.66%) (Table 3 and Table 4).

In the present study, the 10 ISSR primers showed high levels of polymorphisms when compared to those revealed using different molecular markers on the same species, such as RAPD and allozymes analysis [6,14]. When comparing ISSR markers, the genetic diversity observed in populations of argan trees found within Tunisia is relatively similar to that of argan trees in Morocco [16,17,18,20]. Mouhaddab et al. [17] showed that the genetic diversity based on Nei’s distance was between h = 0.153 and 0.233, which is still lower than the genetic diversity found between the different Tunisian sites. However, if considering all the provenances that were studied as a single source, a high genetic diversity is noted (h = 0.353) which is almost similar to Nei’s genetic diversity found between Tunisian argans. In a different study on argan trees in Morrocco, Pakhrou et al. [20] used ISSR and IRAP markers among 24 populations of the *A. spinosa* and showed a lower Nei’s genetic diversity in all the populations studied compared to the result found in the present study. A third study [15] on Argan trees in Morrocco with ISSR markers showed a Nei’s genetic diversity index (H) that ranged from 0.12 to 0.24 which is also lower than the genetic diversity index in our study. However, if considering all the provenances as a single source, a high genetic diversity is noted (h = 0.35), which is similar to our results.

### 3.3. Genetic Differentiation and Gene Flow

Genetic variability parameters showed high levels of genetic variability among the four provenances of *A. spinosa* in Tunisia, an unsurprising result for an endangered species characterized by small populations [40,41].

Multi locus analysis estimated from 116 loci showed diversity within the four provenances studied; (Gst = 0.17; Nm = 2.42), which is considered lower than the average genetic differentiation coefficient (Gst) among populations of *A. spinosa* in Morocco which indicated high genetic differentiation (Gst = 0.40), (Gst = 0.20), and (Gst = 0.54) [16,17,20]. The differentiation coefficient foundin argan trees is also lower compared to other forest species, for example sessile oak was sited with a differentiation index of Gst = 0.026 [42], *Pinus sylvestris* with Gst = 0.3965 [43]; *Cupressus gigantea* (Gst = 0.36) [44]; *Machilus thunbergii* (Gst = 0.4118) [39], and *Rheum palmatum* and *Rheum tanguticum* (Gst palmatum = 0.537; Gst tanguticum = 0.497) [45]. This may be a result of the ecological differences among the two countries or the limited number of samples compared to the studies in Morocco.

An ANOVA analysis has confirmed a high genetic variability within provenances, showing that 18% of the total variance was between populations while 82% was within populations. The genetic variability of argan could be explained by various factors, including their geographical localization, soil composition, and climatic changes between sites, and their breeding system [46].

We observed a genetic link between “H” Souss and Korbus “A” (Souss plain), while the two other sites were detached forming separate groups which means that gene flow between those two populations is limited. This genetic diversity can be explained by some factors as geographical barriers, wind direction, and also mode of reproduction as reported by El Mousadik and Petit that they have used allozyme markers (Gst = 0.25) PCR-RFLP and the DNAcp markers (Gst = 0.60) in their study [6]. The genetic structure of plant populations designates the interchange between many diagnostic procedures containing the long-term evolutionary history of the species, genetic mutation, genetic drift, gene flow, and selection [47,48].

In this study, the gene flow in and among sites was high as 2.42 compared to ((Nm = 0.4), (Nm = 1.78) and (Nm = 0.73)) argan populations in Morocco [16,17,18,20]. Gene flow has an important role in the determination of genetic structure. Normally whenever Nm > 1 the prevention of divergence from genetic drift is important [49]. We can then assume that pollen movements are the essential cause of this high gene flow among populations. Results with Nei’s genetic distance showed that the highest genetic variability was among “JB” and “A” sites and a lower gene difference was among “H” and “A”.

### 3.4. Genetic Structure

Spatial genetic structure analysis results showed that all populations were assigned to three groups, as indicated by the phylogenetic tree UPGMA (Figure 4), factorial analysis (Figure 5), and the Bayesian-based approach from the STRUCTURE program (Figure 6). The UPGMA dendrogram results showed that the argan trees used in the present study can be divided into two distinct groups or clusters. The first group is detached from other groups and present the provenence “S” the second group is divided in two subgroups, one is formed with two different provenances which are “A” and “H” which suggest that the geographic distribution is not the principal factor shaping the present populations’ genetic structure, the second subgroup is only formed with the provenance “JB” (Figure 5). The hypothesis that can explain these results is that wind and insects are the factors for the reproduction system of the argan tree pollen scattering mechanism [5,23]. The similar result was observed in the factorial analysis, as we can spot that trees from JB (from 1 to 15) and S (from 45 to 60) are forming individual groups while the two other sites form one group. This result was also confirmed by STRUCTURE program which indicates that the geographical distribution is not the main factor that formed these populations’ genetic structure. Genetic variability of argan is caused by both environmental factors and individual differences. Information about the genetic relation between the four provenances of *Argania spinosa* is important for possible ex situ conservation. The results that we obtained in this study improve our understanding on population structure of the argan tree and give valuable information about management implications for this species, as this diversity among populations should be maintained for later conservation of this species [50].

### 3.5. HRM

In this work, we reported the use of HRM analysis in plants for scanning and genotyping of *A. spinosa* individuals which have not reported before. The HRM analysis of gDNA PCR amplicons is a fast, simple, and cost-effective method for identification of variations of argan individuals. A 130-bp region of the *D6D* gene was scanned by HRM analysis to identify polymorphic variants in 60 argan individuals collected from four different regions of Tunisia. Sixty individuals were grouped into 10 different categories after HRM analysis. The HRM analysis demonstrated that there are large variations among the argan individuals for the *D6D* gene, even if they grow in the same location under similar conditions. Analysis of fatty acid content, especially LA and ALA, supports the HRM results. LA and ALA contents show high variability in argan individuals. The 10 different “groups” must be verified with sequencing of the PCR products to reveal the polymorphisms in an upcoming study, to reveal the polymorphisms.

### 3.6. Fatty Acid Analysis

Omega-3 Fatty acids (omega-3 FAs) are found normally in seafood, some plants and livestock in limited concentrations. Normally fish oils are the only concentrated source of eicosapentaenoic acid (EPA; 20:5 omega-3) and docosahexaenoic acid (DHA; 22:6 omega-3) [51]. Clinical trials proved that EPA and DHA can improve joint pain in patients with rheumatoid arthritis, and also can improve skin lesions, lower the hyperlipidemia from etretinates, decrease the toxicity of cyclosporin in patients with psoriasis, and in various animal models omega 3 fatty acids decrease the number and size of tumors and increase the time elapsed before appearance of tumors [52].

EPA and DHA actually represent a subject of much interest, because of their important roles in human health and nutrition, such as neonatal retinal and brain development, as well as cardiovascular health and disease prevention [53]. EPA is a component of mammalian cell membrane and also a precursor of the eicosanoid. It has important roles as a biological effectors involved in inflammatory responses, blood pressure regulation, blood clotting, and cell signaling [54].

According to the results in this study, the quantity of EPA and DHA is found high compared to some plants from other studies, as proved in many articles EPA and DHA are present in higher quantities than marine organisms such as fish, fish oil, shrimp. However, the quantity present in plants is normally too low, and the content in EPA and DHA has not exceeded the 0.01 g/100 g in the majority of plants which are considered as traces. Another study on Purslane leaves showed that it contains a quantity of EPA and DHA of about 0.36 g/100 g in leaves [55] which presents a very interesting quantity for plants. Later in 1996, a study concerning fatty acid percentage contents in edible wild plants was undertaken treating different plants; *Beta maritima* L., *Cakile maritima* Scopoli, *Portulaca oleracea* L., *Amaranthus viridis* L., *Chenopodium album* L., *Sonchus oleraceus* L. and *Stellaria media* Villars, *Crithmum maritimum* L., *Malva sylvestris* L., *Parietaria diffusa* Mert., *Pichris echioides* L., *Rumex crispus* L., *Salicornia europaea* L., *Sisymbrium irio* L., *Sonchus oleraceus* L., *Sonchus tenerrimus* L., *Stellaria media* Villars, and *Verbena officinalis* L. In this study, some of these mentioned plants scored the presence of EPA and DHA. The content in EPA in these different plants went from 0 to 0.9 g/100 g, the content of DHA went from 0 to 2.3 g/100 g [56], however, this present study showed that the content in EPA and DHA in argan tree leaves is considered very important compared with other plants, and what make it more interesting is that argan tree is an evergreen tree with no valorized leaves.

The main known sources of EPA and DHA are different kinds of seafood (Mackerel 1.8–5.3 g/100 g, Herring 1.2–3.1 g/100 g, Salmon 1.0–1.4 g/100 g, Tuna 0.5–1.6 g/100 g, Trout 0.5–1.6 g/100 g, Halibut 0.4–0.9 g/100 g Shrimp) [57]. Additionally, in another study, shrimp in many size categories also supplied 0.2 g/100 g of EPA plus DHA [58]. Compared to these studies, the content of EPA and DHA in agran trees in Tunisia is considered high and even comparable the content of EPA and DHA in seafood. This particular study highlights the richness of argan leaves which make it a great candidate for pharmaceutical or cosmetic products.

### 3.7. Correlation between the Different Analyses

**Figure 8:**
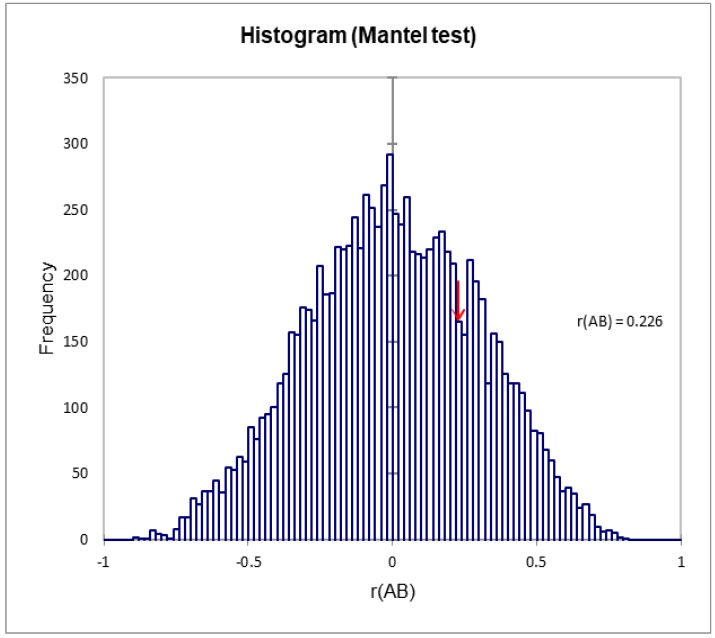
Mantel test between geographical distance and genetic distance among different populations of *A. spinosa.*

The Mantel test was used to calculate the correlation between the genetic and geographical distances. Figure 8 shows that there was no significant correlation between geographical distance and genetic distance (r = 0.226, *p* = 0.481, alpha = 0.05). This indicates that the great majority of the genetic variation resided within populations.

**Figure 9:**
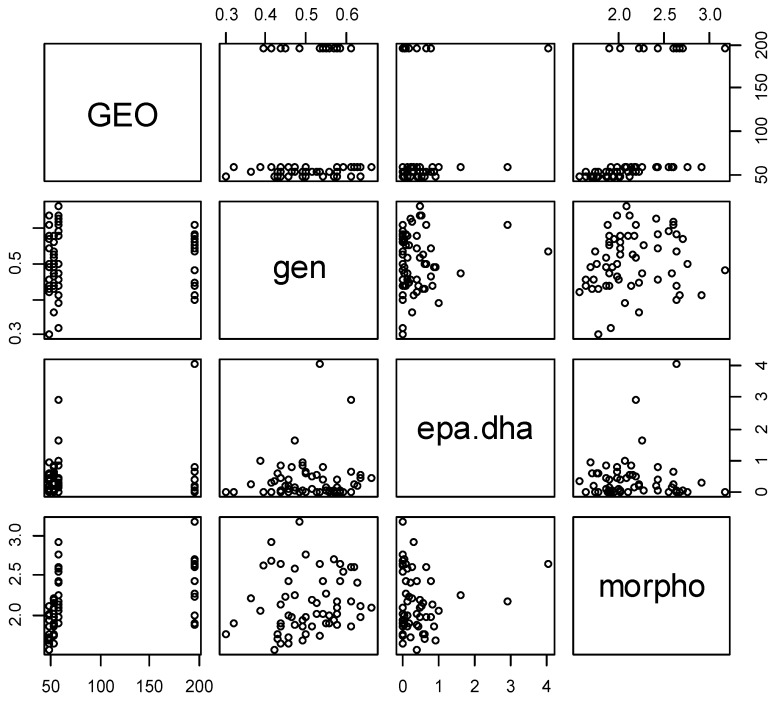
Correlations between the different matrices (geo: geographic matrix; gen: genetic matrix; epa.dha: EPA and DHA content in leaves matrix; morpho: morphological matrix).

A correlation test was conducted using Rstudio software [59] to determine the correlation between different factors (Figure 9). The only significant positive correlation was shown between the genetic and morphological factors. None of the matrices had a significant correlation with the geographical matrix. The significance of correlation of two matrices (genetic and morphological) may suggest that there is some linkage between the ISSR marker used and the morphological characters chosen. As shown by Reddy et al. (2002) [60], ISSR markers were successfully used for gene tagging or used in marker-assisted selection for agronomic traits which also represents many of the characters chosen in this work.

## 4. Materials and Methods

### 4.1. The Geographical Area of the Study

**Figure 10:**
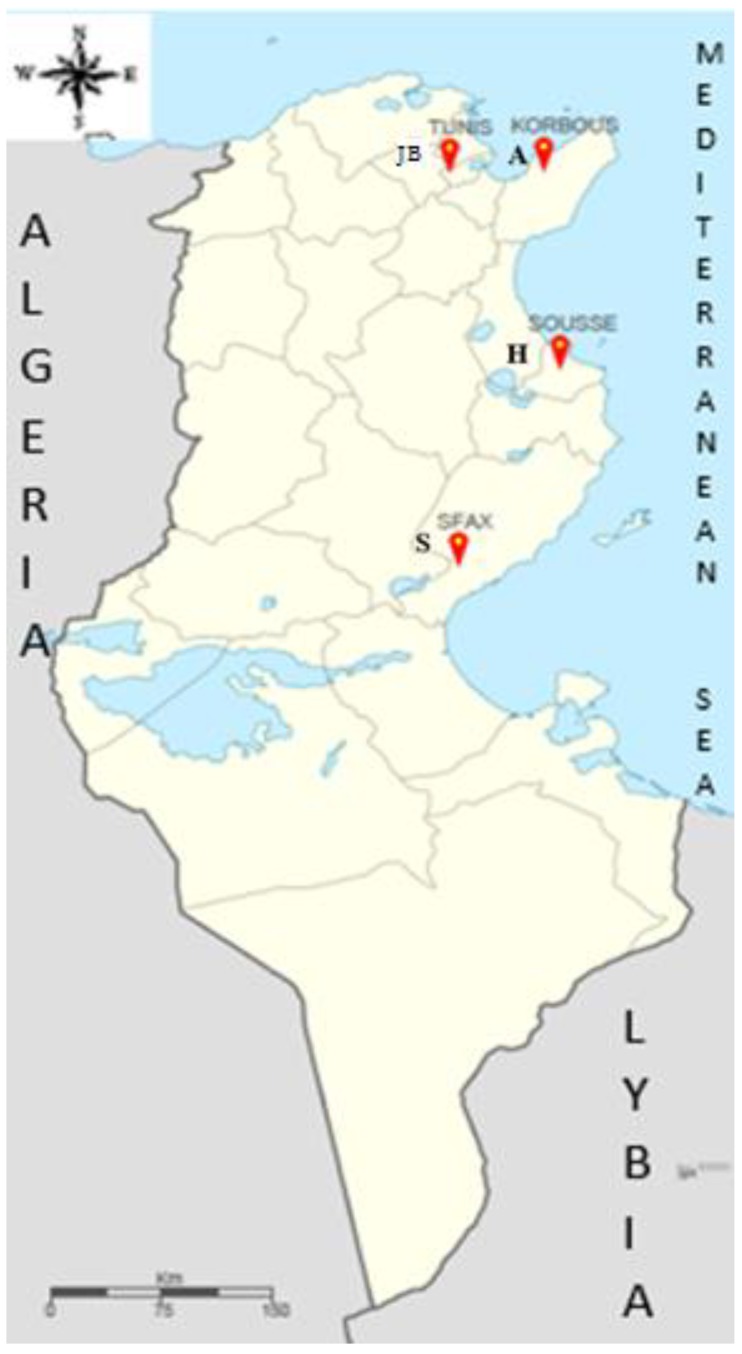
The geographical distribution of the sites in Tunisia.

The samples of leaves and fruits used in this study were collected from 60 trees located in four different sites in Tunisia (Figure 10, Table 7) (Sfax, Souss, Korbous, Tunis (Botanical garden)). These sites are located in four different cities that have different climatic conditions in regards to the altitude and different composition of soil (Table 7). The trees growing in the botanical garden were planted in the 1960s and are from Moroccan origins from many provinces in Morocco [61], while the other sites are from unknown origins.

### 4.2. Morphological and Pomological Characterization

Fifteen trees from each site were chosen to carry out this study. The assessment of morphological variability was based on the observation and measurement of morphological characters related to leaves, fruits, seeds, kernels, flowers, and the general appearance of selected trees.

Several characters were chosen according to the Avocado descriptor (UPOV Code: PERSE_AME, 2010) since there is not yet a descriptor dedicated to argan trees. Morphometric analysis is essential to make a first “inventory” to identify the most discriminating and informative characters in the description of phenotypic variability and its organization. Sixty argan trees were analyzed based on quantitative and qualitative morphological characters (Table 8).

Ten fruits from each of the 60 trees were collected from different levels and positions of the tree to measure the pomological characters. The sample collection was made between June and August since argan fruits do not reach maturity at the same time. The fruits were pitted to separate the pericarp from the seeds. A morphological study was carried out on the seeds. Subsequently, the kernels of each seed were removed, and a measure of the selected kernel-related traits was established (Table 8).

### 4.3. Extraction and Purification of DNA

The genomic DNA was extracted from 60 trees, using a modified and optimized CTAB protocol [62]. Difficulties have been faced in isolating DNA free from contaminating proteins and polysaccharides since leaves from argan are too rich with polyphenols, tanins, and polysaccharides [63]. That is the reason why a washing step was added just after grinding leaves in liquid nitrogen and before starting with the modified CTAB protocol, which consisted of washing the grinded leaves at least five times with 1 mL of the washing solution (1M Hepes, 0.1% PVP and 4% βmercaptoethanol), until the supernatant became clear. After the washing step, the extraction protocol began with the addition of 1 mL of 2% CTAB extraction buffer (1 M Tris-HCl pH 8.0, 1.36 M NaCl; 0.5 M EDTA pH 8.0, 0.5 M Poly-Vinyl Pyrrolidone (PVP) and CTAB 2%). The tubes were then incubated at 65 °C for 60 min. Chloroform:isoamyl alcohol (24:1) was used later to extract genomic DNA, centrifugation was done at 4 °C (13.000 rpm for 12 min). To precipitate the DNA, isopropanol (2/3 volume of supernatant) was added and incubation was done for at least 1 h at −20 °C. A centrifugation at 14,000 rpm for 15 min was later performed to collect precipitated DNA. The DNA was washed by 75% ethanol. The final step consisted in drying the pellet and adding TE buffer. A classical RNAse treatment was later performed for better quality of DNA. The quality and quantity of DNA obtained were checked by nanodrop and by electrophoresis in 0.8% agarose gel containing ethidium bromide, respectively. The DNA was stored at −20 °C for later use.

### 4.4. ISSR Analysis

In this study, 10 ISSR primers that produced high polymorphism were used to estimate the genetic variability of 60 samples. These primers present di- and tri-repeat motifs (Table 2), previously described by Souto Alves et al. [64] that were used to check the diversity of a wide range of forest species. For example, *Cassia fistula* L. [24], *Murraya koenigii* [65], *Apterosperma oblata* [66]. The amplification of DNA by PCR was performed in a thermocycler (Biorad, T100, USA). Temperatures that gave a clear pattern were then repeated until the best Tm was selected. The MgCl_2_ concentration and annealing temperature varied according to the primers. The PCR reactions were performed in 25 μL reaction mixture containing 50 ng of genomic DNA, 1× PCR buffer Taq polymerase buffer, Taq polymerase (1 U), 0.2 mM of dNTP, and 0.2 mM of primers. The concentration of MgCl_2_ depends on the nature of the primers. The program performed starts with an initial denaturation cycle at 94 °C for 5 min; followed by 30 cycles of 30 s denaturation at 94 °C, annealing at 47–60 °C for 30 s (depending on the type of primers) and 45 s extension at 72 °C; and ending with final extension cycle at 72 °C, for 10 min. The PCR products were visualized by 2% agarose gel.

### 4.5. PCR and HRM Conditions

HRM reactions were carried out in two rounds to detect delta-6-desaturase (*D6D*) variants in 60 *A. spinosa* individuals. In the first round, *D6D* (Accession number: AY131238.1) was amplified using 20 ng genomic DNA with primers *D6D*-F1:5′-CGATTCTTGGGAAAGCCTATGA-3′ and *D6D*-R1:5′-CATGTAGAGGCAGGATGGAATG-3′. PCR reactions included 0.4 µM forward and reverse primers ((0.25 mM dNTPs, 2 mM MgCl_2_ and 1 U GoTaq DNA polymerase (Promega, San Luis Obispo, CA, USA)). PCR cycling conditions were programmed as initial denaturation at 95 °C for 10 min, followed by 35 amplification cycles of denaturation at 95 °C for 15 s, annealing at 60 °C for 15 s, and elongation at 72 °C for 15 s with a final extension at 72 °C for 5 min in a thermal cycler (Bio Rad, T100, Hercules, CA, USA). Resulting PCR products were analyzed in 1.2% agarose gels. In the second round, HRM-PCR reactions were performed in a Light Cycler 480 II (Roche Applied Sciences, Indianapolis, IN, USA) and first-round PCR products, diluted 50 fold, were used as DNA template in HRM-PCR reactions. HRM-PCR reactions were performed in a final volume of 10 µL containing 1× the Luminaris Color HRM qPCR Master Mix (Thermo™, Waltham, MA, USA), 0.25 µM of each internal primer, and diluted first round PCR products. Amplification and melting conditions were as follows: 95 °C for 10 min, 35 cycles at 95 °C for 15 s, 60 °C for 15 s, 72 °C for 15 s, and melting at a gradient from 60 to 95 °C at 0.02 °C per second, 25 fluorescence acquisitions per 1 °C [67]. Two technical replicates were used in all reactions and data was analyzed by using Gene Scanning Program Ver. 1.5.

### 4.6. Fatty Acid Analysis

Total lipids were extracted from lyophilized leaves. After the 24 h rotary extraction with petrolium ether, methyl esters were analyzed [68]. The analysis of fatty acid methyl esters was performed by using gas chromatography (GC) (Perkin-Elmer Claurus 500 GC, Autosystem XL, FID, GC Software Turbochrom 4.1, Awenud-Shelton, DE, USA). GC conditions were as follows: Column SGE BPX70 (SGE Analytic Science, Australia) 60 mm × 0.25 mm ID × 0.25 μm, injection volume 1 μL, injection temperature 220 °C, air 450 mL/min, H_2_ 45 mL/min, flame ionization detector (FID) temperature 240 °C and column temperature, 120 °C for 5 min, programmed at 5 °C increments per min up to 240 °C for 15 min (total program process time was 45 min). Fatty acid methyl esters were identified by comparison of their retention times with a known reference material (Certificated Argan oil) and authentic standards (Supelco 37 Component FAME Mixture, 47885-U Supelco). Two replicate GC analyses were performed, and the results of fatty acid methyl esters were calculated as %. Fatty acid as mg/100 g dried leaves were calculated according to Equation (1):

Fatty acid (mg/100 g): (the percent value of fatty acid × fatty content (g/100 g))/100(1)

### 4.7. Statistical Analysis

The morphological data was analyzed by different statistical procedures of Rstudio software [69] and XLSTAT software version 2014.5.03 (XLStat software; Addinsoft, Paris, France). ANOVA was performed by XLSTAT software version 2014.5.03. This analysis makes it possible to compare the averages of the parameters measured in several populations [69,70]. It defines the difference among individuals and the residual variation within and between the groups of individuals studied. Thus, the comparison of the variances allows us to conclude on the difference among the intra- and inter-population variance. The degrees of freedom are calculated according to the following Equations (2) and (3) [3]:

k1 = (p − 1),(2)

k2 = p (n − 1) with p: number of populations and n: number of individuals.(3)

Furthermore, a joint analysis of all the parameters was applied to study the morphological variability and its structuring by performing a principal component analysis (PCA) for the quantitative variables. A classification dendrogram was constructed according to Ward’s method based on gower distance all with the software Rstudio. Genetic determinism of ISSR primers is dominant. The amplified fragments on the agarose gel, presented bands in different level according to their molecular weight (bp) the bands were scored using a binary code (present (1) or absent (0)). The exact location of bands and the exact molecular weight were determined with gelpro32 (Syngene, Inc., San Diego, CA, USA). Each of these fragments represents a locus. Data was compiled in a 0/1 binary data matrix of 60 individuals using MS Excel. The parameters related to the genetic variability in and between sites (i.e., the percentage of polymorphic bands (PPB), Nei’s gene diversity (h), Nei’s genetic differentiation index among populations (Gst), allelic richness (A), and gene flow (Nm)) were estimated using POPgen 1.32 [71]. An estimate of Nm among populations was calculated with the formula of Nm = 0.5 (1–Gst)/Gst [72]. The genetic distance matrix was employed to construct a UPGMA dendrogram based on genetic distances calculated with Maximum Composite Likelihood in Popgene ver. 1.32 [73] and imported to MEGA6 [74]. The software Darwin version 6.0.10 [75] was used for factorial analysis and again for another version of UPGMA which we used eventually in this present study. The molecular variance (AMOVA), using GenALEX version 6.5b3 [76], was used to estimate the distribution of the genetic variation between and within the different provenances. A Bayesian analysis of ISSR population structure on the entire data set, using STRUCTURE 3.2 [77], was used to test for genetic admixture across provenances. STRUCTURE algorithm was run using the basic model with admixture and correlated allele frequencies, with the assumed number of genetic K clusters varying from 1 to 10 (the total number of provenances), 10 replicate runs per K value (number of populations), and the burn-in period and Markov Chain Monte Carlo (MCMC) set to 500,000 and 5,000,000 iterations, respectively. To provide a fast way to assess and visualize likelihood values across multiple values of K for easier detection of the number of genetic groups that best fit the data, the program ‘structure harvester’ was used [78].

A Mantel test was performed to determine the correlation between the genetic distance [79] and the geographical distance among the populations using the XLSTAT.

A correlation test was conducted using Rstudio software [59] to determine the correlation between the different factors.

## 5. Conclusions and Perspective

In order to study the morphological, molecular, and biochemical diversity of argan trees in Tunisia, 60 trees were selected for this study, and leaves and fruits from each tree were collected. With regard to edaphic conditions, the argan tree has no requirement for soil type, based on the results obtained.

In the first part of this study it appears that the argan tree normally develops in different types of soils (sandy and loamy), tolerates a wide pH range from 4 to 8.3, and a high concentration of limestone. The argan tree is a thermophilic and xerophilic tree, whose range overlaps with both humid and arid bioclimates. The introduction of this species into reforestation programs offers a sustainable reforestation solution in the arid and semi-arid zones and also in those affected by salinity and would therefore allow diversified exploitations. For the morphological characterization part, results have shown the existence of different forms, colors, and sizes of fruit and leaves. The results obtained from ANOVA showed that some are more discriminating than others which allowed to subdivide the 60 argan trees in three groups. Principal component analysis (PCA) showed that individuals belonging to the four sites exhibit considerable morphological variability, taking into account quantitative and qualitative variables. The results obtained from Ward’s dendrogram showed a classification of argan trees studied. These results indicated the presence of substantial morphological variability at the intra- and inter-site level. These groups could be dependent on the geographical origin, and we noticed also a formation of three groups; trees from Sfax and Sousse overlapped in same group while trees from Botanical garden and Korbous formed individual groups, although some detachment of some individuals from their original sites was observed.

In the second part of this study, a great level of genetic variation among ISSR markers was shown, as a result of this study, proving that ISSR was an efficient method to assess the genetic differentiation in argan trees. A high amount of genetic diversity within argan populations was shown by AMOVA analysis, which indicates that every site is very important for the argan tree gene pool. The results with UPGMA, factorial analysis, and STRUCTURE analyses divided all the individuals into three groups; one group contains individuals from both sites Korbous and Sousse the two other sites formed individual groups. This result did not correlate with the morphological characterization results, which leads to the conclusion that the morphological diversity determined by ISSR is not related to the morphological variability determined with the chosen variables.

In the third part of this study, HRM was performed to detect *D6D* gene polymorphisms, which divided the 60 individuals to 10 groups. We could not correlate this result to the results of molecular diversity with ISSR or with the other parts of this study. In fact, each study divided the samples into different groups. Conservation strategies should be planned to preserve this high genetic diversity within populations and to prevent potential extinction of this important species in Tunisia.

Based on our results we suggest possible strategies for conservation of argan trees. In the last part, fatty acid analysis by gas chromatography showed that argan tree leaves are rich in omega 3 and 6, and more importantly in eicosapentaenoic acid (EPA) and docosahexaenoic acid (DHA) which is interesting since normally plants do not produce EPA or DHA. Both EPA and DHA quantities produced in some trees are even more than that produced in salmon fish or comparable to herring fish which are known for its high concentration of EPA and DHA, so this result can be revolutionary. In view of these results, it would be interesting to grow these particular trees that scored the highest quantity of EPA and DHA, in order to develop a genetic selection that would increase the ratio of EPA/DHA.

Argan trees in Tunisia are not valued or exploited in any field, and they are totally neglected, contrary to argan in Morocco which are overexploited.

- The first most important management practice is to increase awareness about the importance of this species and start exploiting these trees with moderation especially trees with high genetic diversity. At the same time, we should allow populations to increase in size through natural regeneration and also try to grow argan trees in different locations in Tunisia, especially locations under the threat of desertification since it tolerates high temperatures and salinity and fights desertification.
- The second possible strategy is to focus on the establishment of an ex situ conservation program by seed storage from all the four sites to preserve most of the genetic variation.
- The third possible strategy consists of building a gene bank with a large number of plantations in a natural reserve, taking into consideration all the populations and genetic groups so that we can increase the possibility of gene exchange and ensure long-term conservation of this genetic variation. Knowing the economical, ecological, nutritional, and pharmaceutical value of this tree, it is important to increase awareness in Tunisia about the importance of this tree in order to preserve it. In addition, it would be interesting to combine the studies on the distribution of genetic diversity with other information such as oil composition and morphological characters per population.

## Figures and Tables

**Table 1:** Results of analysis of variance (ANOVA) made for comparison of all variables chosen.

Codes	DF	Sum of sq	Average of sq	F	*P* > F
LF	11	17.398	1.582	6.297	<0.0001
LgF	11	2.305	0.210	1.803	0.080
L/LgF	11	2.168	0.197	7.453	<0.0001
P	11	184.492	16.772	3.955	<0.0001
Ln	11	2.590	0.235	9.305	<0.0001
DF	11	30.207	2.746	2.817	0.006
L G	11	8.328	0.757	4.331	<0.0001
LgG	11	1.236	0.112	1.616	0.124
L/LgG	11	2.043	0.186	2.608	0.011
PGf	11	11.180	1.016	1.919	0.060
PGs	11	5.750	0.523	1.433	0.189
DG	11	5.937	0.540	1.387	0.210
TG	11	1.017	0.092	2.611	0.011
Nb A	11	1.140	0.104	1.356	0.224
Pd A	11	0.048	0.004	1.205	0.309
LA	11	5.211	0.474	3.717	0.001
Lg A	11	0.287	0.026	0.711	0.722
L/Lg A	11	3.445	0.313	2.725	0.008
Ep A	11	0.037	0.003	0.944	0.508
Lfu	11	20.742	1.886	1.928	0.059
LgFu	11	0.473	0.043	1.604	0.128
SF	11	9.696	0.881	1.721	0.097

**Table 2:** List of 10 inter-simple sequence repeat (ISSR) primers, annealing temperatures (Ta), MgCl_2_ concentration optimized for PCR amplification, and length of the amplification products.

Primers	Motif	Ta (°C)	MgCl_2_ (mM)	Size (bp)	TNB	NPB	PPB%	PIC	No. of Bands in Each Population			
									JB	A	H	S
ISSR 1/8	(AG)8C	50 °C	2	200–1000	10	8	80%	54%	10/10	10/10	10/10	10/10
ISSR 3/8	(GA)8YG	50.3 °C	1.5	200–900	8	6	75%	47.6%	8/8	8/8	8/8	8/8
ISSR 4/8	(AC)8YG	49 °C	2	300–1500	14	14	100%	66.4%	14/14	14/14	14/14	14/14
ISSR 6/8	(AC)8YT	52 °C	1.5	300–2500	22	21	95%	78.1%	20/22	19/22	22/22	21/22
ISSR 7/8	(AGC)6	60 °C	2	200–1000	6	5	83%	69.5%	6/6	6/6	5/6	6/6
ISSR 8/8	(CTC)6	57 °C	2	400–2000	11	10	90%	69.9%	10/11	11/11	10/11	10/11
ISSR 807	(GA)8C	48.5 °C	1.7	300–1000	8	6	75%	54.6%	8/8	8/8	8/8	8/8
ISSR 808	(CT)8A	50.8 °C	2	400–2000	11	10	90%	77.1%	11/11	11/11	10/11	10/11
ISSR 857	(AC)8G	53 °C	2	500–2000	13	12	92%	67.7%	13/13	13/13	12/13	13/13
FLA 10	(GA)8CT	50.3 °C	1.8	300–2000	13	12	92%	70.4%	13/13	12/13	11/13	12/13

Y: pyrimidine C or G; Ta: Temperature of annealing; TNB: Total number of bands; NPB: Number of polymorphic bands; PPB%: Percentage of polymorphic bands; JB: botanical garden; A: Korbus; H: Sousse; S: Sfax.

**Table 3:** Intra-population diversity measures of *Argania spinosa* based on ISSR data.

	Mean± Standard Deviation					Polymorphic Loci	
Provenance	Size	na	ne	H	I	NPL	PPL%
JB	15	1.81 ± 0.39	1.44 ± 0.33	0.26 ± 0.18	0.396 ± 0.25	94	81.03%
A	15	1.80 ± 0.40	1.46 ± 0.35	0.27 ± 0.18	0.41 ± 0.25	93	80.17%
H	15	1.77 ± 0.41	1.41 ± 0.35	0.25 ± 0.18	0.38 ± 0.25	90	77.59%
S	15	1.8 ± 0.39	1.43 ± 0.35	0.26 ± 0.17	0.40 ± 0.24	94	81.03%
Total	60	1.90 ± 0.31	1.53 ± 0.32	0.32 ± 0.16	0.47 ± 0.21	104	89.66%

na: Observed number of alleles; ne: Effective number of alleles; h: Nei’s (1973) [28] gene diversity; I: Shannon’s Information index; Na: number of alleles; Ne: effective number of alleles; H: Nei’s (1973) [28] gene diversity; I: Shannon’s information index; NPL: Number of polymorphic loci; % PPL: Percentage of polymorphic loci.

**Table 4:** Genetic distance of Nei (1978) [29] among provenances of *A. spinosa.*

JB	A	H	S	
0.000				JB
0.272	0.000			A
0.247	0.115	0.000		H
0.234	0.237	0.186	0.000	S

**Table 5:** Analysis of molecular variance (AMOVA) within and between provenances of *A. spinosa.*

Source of Variation	df	SS	MS	VC	% of Variation	*p*-Value
Among Pops	3	233.983	77.994	3.967	18%	>0.001
Within Pops	56	1035.200	18.486	18.486	82%	>0.001
Total	59	1269.183		22.453	100%	

df: Degree of freedom; SS: Sum of squares; MS: Mean of squares; VC: Variance components.

**Table 6:** Grouping of 60 argan variants with high resolution melting (HRM) analysis of the Delta-6 desaturase (*D6D*) gene.

Group Number	Individual	Group Number	Individual
1	A1, A2, A5, A6, A7, A10, A11, A12, H2, H7, H9, S1, S3, S9, S11, S12, S14, P3A2	6	H6, H8, S2, S4
2	A9, A13	7	A14, H13, H14, H15
3	A15, H10, S7, S8, S10, S13, S15, P1A1, P3A6, P5F9, P6H9	8	H12, S6
4	A3, A4, H4, H5, P2F1, P3B8, P3C3, P4A8, P8G10, P9A11, A1JB, A3JB, A6JB	9	H3, H1
5	S5, A8JB	10	H11

**Table 7:** Ecogeographic sites of selected argan trees in Tunisia and soil texture of each site.

Codes	Name of Sites (Cities)	Individuals	Soil Texture	pH of Soil	Climate
JB	Botanical garden (Tunis)	P1A, P2F1, P3A2, P3A6, P3B8, P3C3, P4A8, P5F9, P6H9, P8G10, P9A11, A1JB, A3JB, A6JB, A8JB	Silty	8.3	Semi-arid
A	Korbous (Nabeul)	A1, A2, A3, A4, A5, A6, A7, A8, A9, A10, A11, A12, A13, A14, A15	Sand	6.2	Semi-arid
S	Sfax (Sfax)	S1, S2, S3, S4, S5, S6, S7, S8, S9, S10, S11, S12, S13, S14, S15	Silty sand	6	Arid
H	Souss (Souss)	H1, H2, H3, H4, H5, H6, H7, H8, H9, H10, H11, H12, H13, H14, H15	Clay loam	8.4	Semi-Arid

**Table 8:** Quantitative and qualitative morphological characters of argan trees.

	Code	Morphological Character
Fruit		
1	P F F	Fruit weight
2	L F	Fruit length
3	lg F	Width of the fruit
4	LF/lgF	Ratio length width of the fruit
5	D F	Diameter of the fruit
6	F	Fruit shape
7	C F	Fruit color
8	L N	Nape length
9	PG	Weight of the seed
10	PG S	Weight of the seed after drying in the oven
11	LG	Length of the seed
12	Lg	Width of the seed
13	LG/lg G	Ratio length width of the seed
14	DG	Diameter of the seed
15	TG	Number of sprouting lines
16	CL	Color of the seed
17	Nbr A	Number of kernels per seed
18	PA	Weight of kernel
19	LA	Length of the kernel
20	Lg A	Width of the kernel
21	LA/LgA	Ratio length width of the kernel
22	Ep A	Thickness of the kernel
Tree		
23	Fr Fleurs	Frequency of the flowers
24	Fr BF	Frequency of flower buds
25	Fr Fr	Frequency of fruits
26	Frm de l’arbre	Shape of the tree
27	L de l’arbre	Length of the tree
28	D trc	Trunk diameter
29	Fr ramific	Frequency of ramifications
30	Fr ep	Frequency of spines
Leaves		
31	Lf	Length of the leaf
32	Lf	Width of the sheet
33	Sf	Leaf area
